# Case report: HPV related pelvic retroperitoneal squamous cell cancer of unknown primary presenting as ovary neoplasm

**DOI:** 10.1016/j.ijscr.2024.110528

**Published:** 2024-10-28

**Authors:** Hui Yan, Shao-dan Lin

**Affiliations:** a Department of Obstetrics and Gynecology, The First Affiliated Hospital of Ningbo University, Ningbo, Zhejiang 315010, China; b Department of Gynaecological Oncology, Sun Yat Sen Memorial Hospital, Guangzhou, China

**Keywords:** PRSCC, Case report, Retroperitoneal squamous cell cancer, HPV, P16, CIN, Diagnosis

## Abstract

**Introduction:**

Retroperitoneal tumors (RPTs) of the pelvis are rare and often present asymptomatically. We report a rare case of human papillomavirus (HPV)-related primary retroperitoneal squamous cell carcinoma (PRSCC) that was preoperatively misdiagnosed as adnexal cancer.

**Case presentation:**

A menopausal 59-year-old woman presented with right leg pain persisting for two months. Imaging revealed a heterogeneous lesion in the right adnexal area; however, the uterus and left ovary appeared normal. Laboratory tests showed slightly elevated levels of cancer 125 (CA 125) and squamous cell carcinoma (SCC) antigens. The patient underwent surgical staging for the suspected ovarian cancer. Intraoperatively, the bilateral adnexa and uterus appeared normal. A lesion identified in the right pelvic retroperitoneal cavity was resected and its pathological analysis revealed SCC and cervical intraepithelial neoplasia III (CIN III) and immunohistochemical expression of cyclin-dependent kinase inhibitor 2A (p16) in the cervix. HPV 16 was identified by a polymerase chain reaction (PCR). The patient chose not to undergo any additional postoperative treatment. Her leg pain disappeared the day after the procedure but recurred a year later. A new tumor was detected on computed tomography (CT) in the same area.

**Discussion:**

PRSCC is rare and can be misdiagnosed as a gynaecological neoplasm. HPV can migrate to the retroperitoneal space and act as a carcinogen in this location.

**Conclusions:**

HPV infection may contribute to the development of PRSCC. Complete surgical resection, with adjuvant radiotherapy and chemotherapy, is a viable treatment approach.

## Highlights

• PRSCC is rare and often misdiagnosed as ovarian neoplasm.
• RPTs should be considered for pelvic mass with lower-extremity discomfort.
• PRSCC may be related to HPV infection.

## 1 Introduction

Cancer of an unknown primary site (CUP) is a histologically confirmed metastatic cancer in which the primary tumor remains unidentified. It accounts for 3–5 % of all human cancers [1]. Squamous cell carcinoma (SCCs) accounts for 5 % of the CUP cases [1]. Various rare benign tumors and malignant neoplasms, either primary or metastatic, can occur in the retroperitoneum [2,3]. Sarcomas constitute one-third of all malignant retroperitoneal tumors (RPTs), with SCCs representing a tiny fraction. Primary retroperitoneal SCCs (PRSCCs) are extremely rare and often present later in life. Their anatomical location is typically close to vital structures such as major pelvic vessels, leading to diagnostic challenges and therapeutic difficulties [2,3]. The etiology and pathogenesis of PRSCC remain poorly understood, and no standard treatment has been established to date. Some studies have reported detecting human papillomavirus (HPV) infection in heterogeneous retroperitoneal tumors [[4], [5], [6]], although more robust evidence is required to confirm the association between PRSCC and HPV. To enhance the clinical practice guidelines, we reviewed previously reported cases of PRSCC.

## 2 Presentation of case

**Fig. 1:**
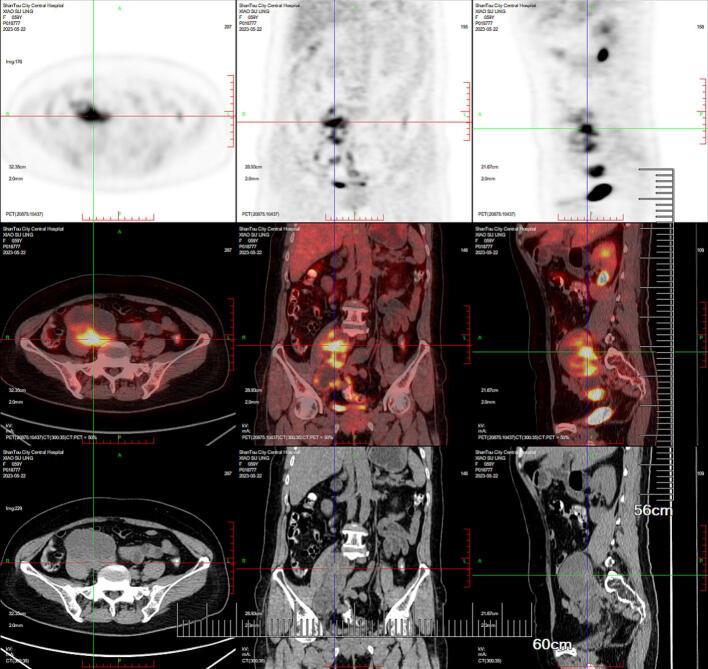
Positron emission tomography/computed tomography (PET/CT) shows a 7 × 6 cm heterogeneous mass in the right adnexal area.

A 59-year-old female presented with right leg pain persisting for two months and walking difficulty. She had no relevant medical or surgical histories. General physical examination results were unremarkable. The uterus was normal in size, and the cervix was smooth. Laboratory tests revealed an SCC antigen level of 4.82 ng/mL (normal ≤2 ng/mL), cancer antigen 125 (CA 125) level of 51.1 U/mL (normal ≤25 U/mL), cancer antigen 19-9 (CA 19-9) level of 40.0 U/mL (normal ≤25 U/mL), and carcinoembryonic antigen (CEA) level of 27.7 ng/mL (normal ≤5 ng/mL). Computed tomography (CT) of the abdomen and pelvis, along with magnetic resonance imaging (MRI) of the pelvis, showed a 7 × 6 cm heterogeneous mass in the right adnexal area. Gastroscopy and colposcopy revealed no dysplasia or malignancy. Fluorodeoxyglucose positron emission tomography (PET) did not reveal any additional areas with increased glucose metabolism, ruling out primary carcinomas or metastatic sites beyond the right pelvic mass (Fig. 1). The ThinPrep cytological test results were normal; however, the test for low-risk HPV was positive. Based on these findings, stage IA ovarian cancer was suspected. An ovarian cancer multidisciplinary team assessed the case base on these result before the surgery.

**Fig. 2:**
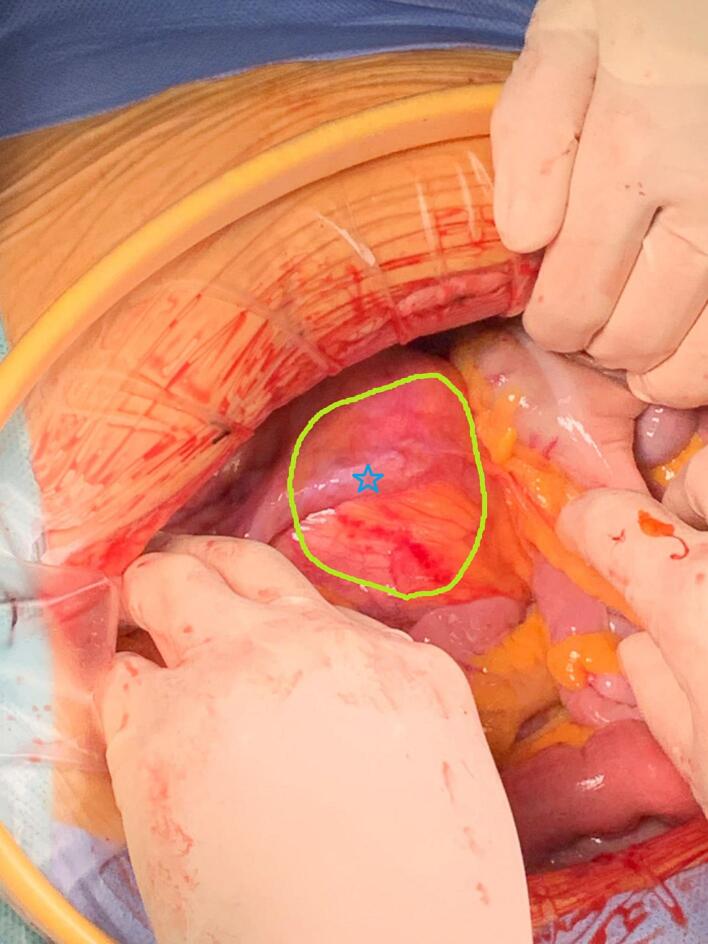
A lesion (yellow circle) was observed in the right pelvic retroperitoneal cavity, above which was the right ureter (blue star), identified before the retroperitoneum was opened. (For interpretation of the references to colour in this figure legend, the reader is referred to the web version of this article.)

**Fig. 3:**
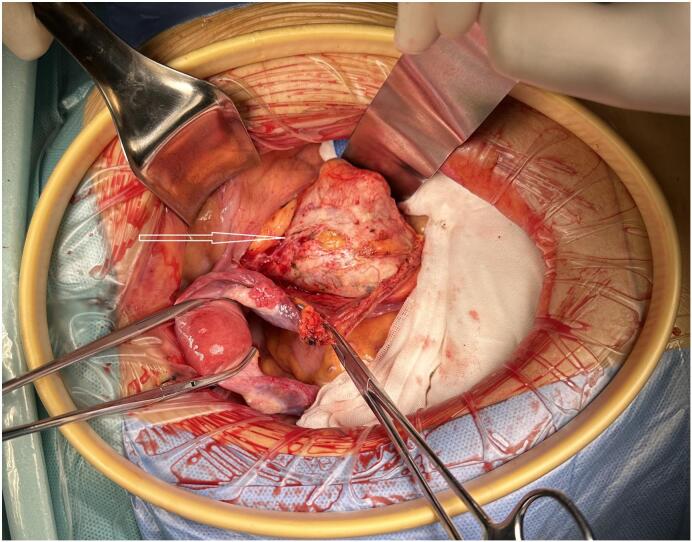
A cystic-solid tumor (arrow) located in the right pelvic retroperitoneal cavity was identified after opening the retroperitoneum.

**Fig. 4:**
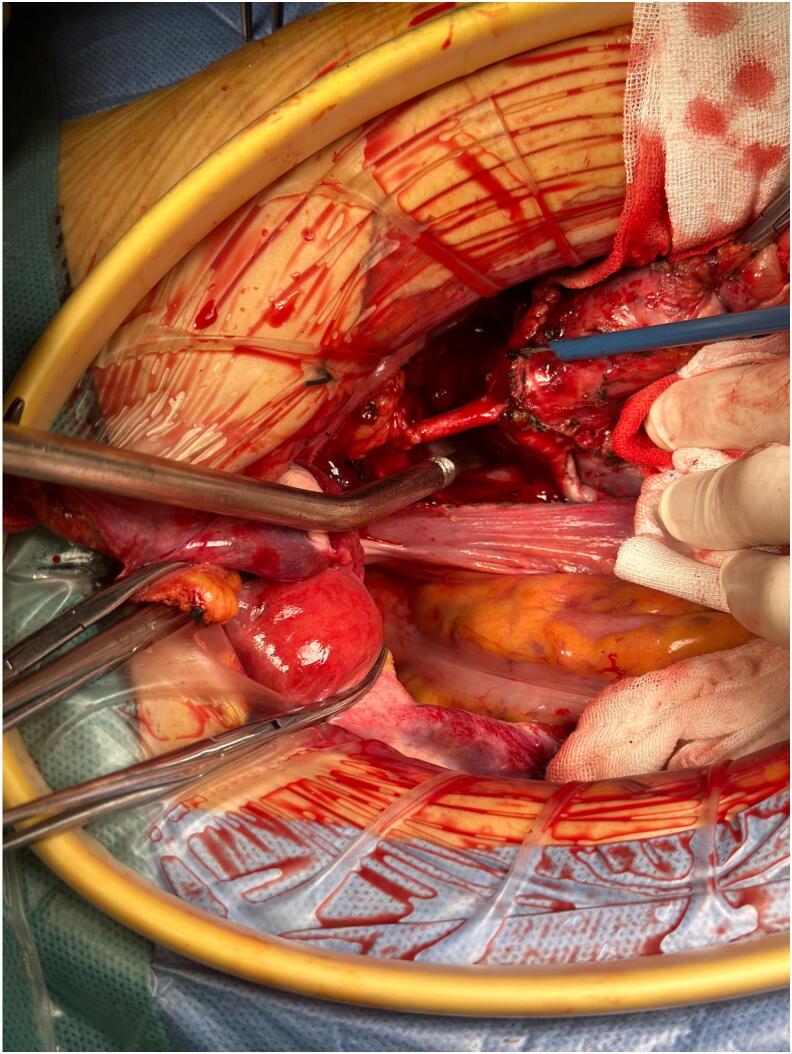
The retroperitoneal lesion compressed the external iliac artery and vein, invaded the right psoas major muscle.

Exploratory laparotomy for staging of the suspected ovarian cancer revealed a normal uterus, smooth cervix, and bilateral adnexa. However, a 7 × 6 cm lesion was identified in the right pelvic retroperitoneal space, with the right ureter positioned above the tumor (Fig. 2). No signs of ovarian disease were observed, and the appendix and upper abdomen appeared normal. The cystic, solid lesion, located in the right pelvic retroperitoneal cavity (Fig. 3), was adherent to the right pelvic wall, compressing the external iliac artery and vein and invading the right psoas major muscle (Fig. 4). The inner portion of the mass was necrotic. It was closely attached to the internal iliac vessels, with the obturator nerve running centrally through it (Fig. 4). Complete tumor was resected, along with total hysterectomy, bilateral adnexectomy, and bilateral pelvic lymphadenectomy. Postoperatively, the patient's leg pain disappeared, and she regained normal walking ability. The surgery was successful without any complications.

**Fig. 5:**
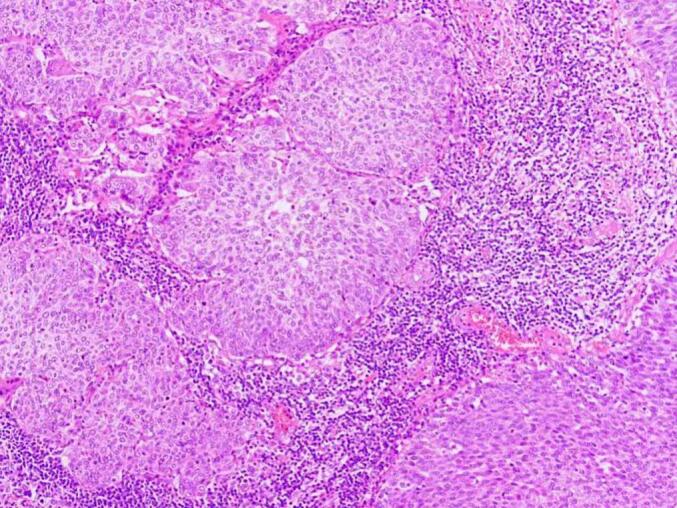
Pathological analysis of the mass showing squamous cell carcinoma (hematoxylin-eosin staining, ×100 magnification).

**Fig. 6:**
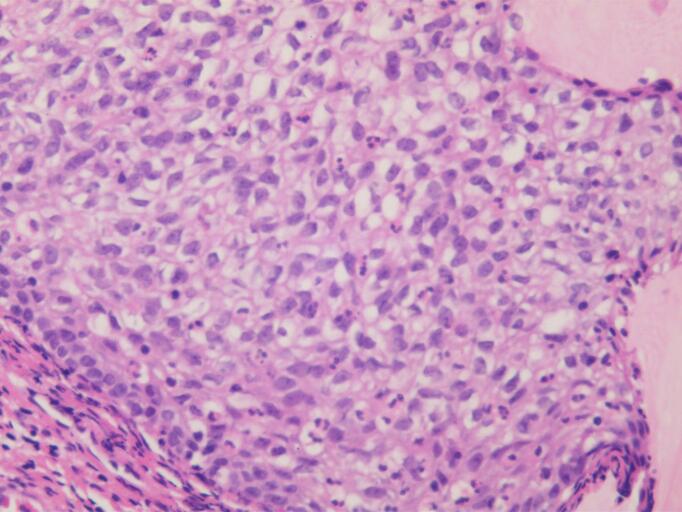
Cervical intraepithelial neoplasia III (CIN III) was identified in the cervix (hematoxylin-eosin staining, ×100 magnification).

**Fig. 7:**
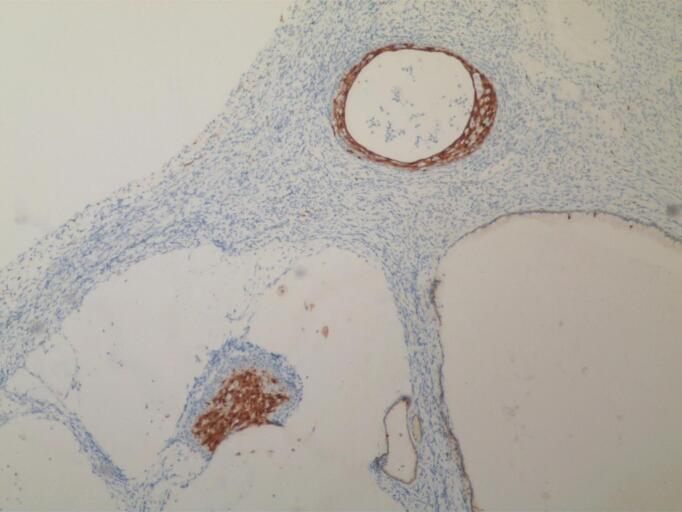
Cyclin-dependent kinase inhibitor 2A (p16) positivity was observed in the cervix.

Frozen sections of the RPT revealed low-grade tumor differentiation, with uncertainty between Brenner tumor and urothelial carcinoma. A ureteroscopy performed by urologists showed a normal bladder and ureter. Pathological analysis revealed SCC (Fig. 5), with cervical intraepithelial neoplasia III (CINIII) detected in the cervix (Fig. 6). All other tissue samples tested negative for malignancy. Immunohistochemistry of the cervix was positive for the cyclin-dependent kinase inhibitor 2A (p16) (Fig. 7). Additional immunohistochemical staining for transformation-related protein 63 (p63) revealed 60 % nuclear positivity in the focal lesions with squamous differentiation. HPV 16 was identified among the 23 surveyed HPV types (17 high- and 6 low-risk) using polymerase chain reaction.

The patient was finally diagnosed with PRSCC and received adjuvant chemoradiotherapy but opted not to undergo further treatment. She returned a year post-surgery with a recurrence of leg pain and swelling in the same leg. A 6 cm mass was identified at the same location on CT, indicating tumor recurrence. After the patient received 6 cycles of taxane- and platinum-based chemotherapy at 3-week intervals, the mass significantly decreased in size, and her leg pain and swelling subsided.

## 3 Discussion

Approximately 20 PRSCC cases have been reported in the literature. Currently, there is no consensus or guidelines for diagnosing and treating PRSCC. Most cases occur in females, with a median age of 52.5 years (range, 27–78 years) [6,7]. The clinical manifestations of PRSCC are diverse and include leg, hip, pelvic, and anal pain, upper thigh numbness, and constipation. Tumor markers are not sensitive to PRSCC detection. Normal tumor marker levels were reported in two cases [4,8], whereas elevated SCC antigen levels (2.3–11.1 ng/mL) were found in four cases [5,7,9,10].

Despite advances in imaging technology, preoperative diagnosis of PRSCC remains challenging. Ultrasonography is commonly used as a first-line investigation owing to its convenience; however, it cannot accurately determine whether a mass is extraperitoneal. PET/CT is useful in ruling out metastatic tumors. CT and MRI play crucial roles in diagnosing RPTs, providing detailed information about the cancer. Malignant tumors are often located in the retroperitoneum, present with well-defined borders and mass-like appearances, and are frequently associated with displacement or distortion of adjacent organs and structures [11].

However, its pathogenesis remains unclear. Nine patients tested positive for p16 [Table 1]. Among these, one case was positive for HPV 18 [4] and another for HPV 16 [5], HPV of unknown type was positive in one case [6]. HPV 16 was also detected in the present case. Isbell et al. [12] reported a case of CIN III preceding PRSCC. In addition, Agrawal et al. [13] reported a patient whose cervical punch biopsy revealed a high-grade squamous intraepithelial lesion, suggesting that a small primary neoplasm in the cervix may have caused a cell clone to migrate to the retroperitoneal space. A plausible explanation involves the transport of latent HPV from the genital tract to the retroperitoneal space, where the retroperitoneal lymph nodes capture it. Pandey et al. [14] reported that the exact origin of PRSCC is unknown and speculated that the metaplasia of embryonic cell nests could be a potential cause. Additionally, surgical interventions may result in iatrogenic deposition of viruses or tumor cells or potentially cause microscopic lymphatic spread of the virus to the retroperitoneum, leading to secondary retroperitoneal SCC. HPV, captured by the lymph nodes or planted in the cervical region, integrates with the genome of these cell nuclei and activates the transcription of E6 and E7 carcinogenic proteins. The transcription of viral carcinogenic proteins, such as E6 and E7, in tumor cell nuclei causes carcinogenesis by interacting with and degrading the primary host tumor-suppressor proteins.

**Table 1:** Presentation and histology of the 10 cases of pelvic HPV/p16+ SCC of unknown primary origin.

Case	Age	M/F	Complaint	Surgery history	Tumor location	P16/HPV	Treatment	Year and reference
1	34	F	Unknown	None	Right psoas	P16	Biopsy + Cisplatin+ pelvic radiation + multiple chemotherapies and biologically targeted agent	2010, Clements A., et al.
2	27	F	Unknown	None	Left psoas	P16	Biopsy + Taxol or carboplatin + definitive chemoradiation	2010, Clements A., et al.
3	54	F	Unknown	None	Right pelvic lymph node	Hpv + P16	Resection of pelvic and paraaortic adenopathy followed by chemoradiation with carboplatin + radiation + surgical resection	2010, Clements A., et al.
4	56	F	None (health examination)	None	Left retroperitoneal adjacent to the common iliac vessel, obturator nerve, and external iliac vessel	Hpv18	Laparotomy + complete resection of the tumor with left pelvic lymph node dissection. 4 course PF + Planned additional radiotherapy	2015, Oh HJ., et al.
5	50	F	Pain in right leg and hip and difficulty in walking	Cervical punch biopsy revealed HSIL	Right-sided retroperitoneal mass in close contact with right iliac vessels and ureter	P16	Biopsy + chemoradiation + Second line chemotherapy Cisplatin/Topotecan + zometa monthly	2016, Agrawal A., et al.
6	69	F	Left upper thigh numbness and pain	Hysterectomy + BSO	Left retroperitoneal mass	P16	Mass resection + chemoradiation (VAMT + weekly cisplatin)	2016, Isbell A., et al.
7	58	F	Left hip pain	Hysterectomy + BSO, CINIII	Left pelvic side wall	P16	Laparotomy + mass resection cisplatin-based chemoradiation	2016, Isbell A., et al.
8	47	F	Left leg pain	None	Left sided retroperitoneal	P16	Biopsy + no further therapy	2016, Isbell A., et al.
9	76	F	Anal pain	Hysterectomy + BSO	Pelvic cavity	Hpv16 + P16	Laparotomy biopsy + 5 courses TC+ IDS+ 3 more course TC	2019, Matsuzaka Y., et al.
10	59	F	Right leg pain and difficulty in walking	None	Right retroperitoneal Our case	Hpv16	Laparotomy + radical mass resection, hysterectomy, BSO + bilateral pelvic lymph node dissection	2023 Our case

No optimal therapeutic modality for PRSCC has been established. Complete surgical resection is more significant than adjuvant chemotherapy [9]. The removal of all affected organs is essential. Total hysterectomy and bilateral salpingo-oophorectomy (BSO) are often performed to rule out metastasis from a primary gynecologic malignancy [10]. In two previous cases [10,15] and in our case, total hysterectomy and BSO were performed, but histological analysis revealed no tumors in the uterus or adnexa. In contrast, Oh et al. [4] reported a case of complete tumor resection with left pelvic lymph node dissection without hysterectomy or BSO; however, the patient's prognosis remained unknown. Fertility-preserving surgery may be considered for patients desiring fertility contingent on the absence of metastasis on PET/CT, and close follow-up is recommended. Radical hysterectomy with pelvic lymphadenectomy was the only method employed by Chen et al. [10], but requires further verification.

The decision between primary and interval debulking surgery (IDS) remains a subject of ongoing debate. Three cases [4,10,15], similar to ours, underwent primary surgery, whereas IDS was performed in two cases [5,7]. In one case of suspected ovarian cancer, 5 cycles of paclitaxel and carboplatin reduced the tumor size from 14 cm to 6.5 cm, allowing for IDS, which confirmed PRSCC [5]. Another case demonstrated that after 5 cycles of chemotherapy, the PRSCC tumor was significantly smaller, and the involvement of the left iliac artery/vein and ureteral tract had disappeared [7]. The tumor was responsive to chemotherapy, reducing surgical complications. Surgical complications have been reported in three cases, including sacrifice of the external iliac vein [7], removal of the distal ureter owing to segment involvement [10,15], and development of a vesicovaginal fistula post-surgery [15].

Exploratory laparotomy was more often be performed in the PRSCC cases [4,5,9] and laparoscopy was rarely reported. RPT are generally larger and arise in an anatomically complex and surgically inaccessible site with surrounding vital structures limiting wide margins. A complete margin-negative surgical resection is more possibility in the laparotomy regime.

The most common chemotherapeutic regimens for PRSCC include carboplatin and paclitaxel, followed by cisplatin [16]. Carboplatin and paclitaxel regimens were administered in four cases [5,9,17,18]. Cisplatin-based chemoradiation is often used, and many patients undergo multiple chemotherapies. However, there is no consensus regarding the optimal chemotherapy protocol.

## 4 Conclusion

Surgeons should be vigilant for RPTs when encountering a pelvic mass accompanied by symptoms such as lower extremity discomfort, pain, numbness, and swelling. HPV infection is a plausible explanation of PRSCC; however, further studies are required to validate this hypothesis. Next-generation sequencing of tumors may identify genetic mutations and potentially guide more effective treatments. Owing to the rarity of PRSCC, primary surgery with total resection is challenging, making IDS a viable alternative. Total mass resection should preserve organ function and ensure the patient's health. There are no well-established chemotherapy or radiotherapy regimens for PRSCC, both being crucial treatment components. The most effective regimen should be determined based on more case studies.

## Author contribution

Hui Yan: Conceptualization, Data curation, Methodology, Supervision, Visualization, Writing - original draft, Writing - review & editing.

Shan-Dan Lin: Data curation, Investigation, Methodology, Supervision, Visualization, Writing - review & editing.

## Informed consent

All patients gave written informed consent for publication and accompanying images. The editor-in-chief of this journal can review a copy of the written consent form upon request.

## Ethical approval

The Ethical Committee approved the study (2024-002RS).

## Guarantor

Hui Yan.

## Methods

The work has been reported in line with the SCARE criteria [19].

## Research registration number

1. Name of the registry: National Health insurance information platform
2. Unique identifying number or registration ID: MR-33-24-027168
3. Hyperlink to your specific registration (must be publicly accessible and will be checked): https://www.medicalresearch.org.cn

## Funding

This study did not receive specific grants from public, commercial, or non-profit funding agencies.

## Conflict of interest statement

None.
